# The controlling nutritional status score as a predictor of survival in hematological malignancies: a systematic review and meta-analysis

**DOI:** 10.3389/fnut.2024.1402328

**Published:** 2024-06-13

**Authors:** Guimei Lu, Qingqing Li

**Affiliations:** ^1^Department of Laboratory, Liaoning Cancer Hospital and Institute, Cancer Hospital of China Medical University, Shenyang, China; ^2^Department of Endoscopy, Liaoning Cancer Hospital and Institute, Cancer Hospital of China Medical University, Shenyang, China

**Keywords:** nutrition, lymphoma, leukemia, survival, recurrence

## Abstract

**Objective:**

The controlling nutritional status score (CONUT) has been widely used for ascertaining the prognosis of various cancers. However, its use in patients with hematological malignancies remains unclear. This review examined evidence on the utility of CONUT as a prognostic marker for patients with hematological malignancies.

**Methods:**

All cohort studies that examined the association between CONUT and outcomes of hematological malignancies and were published on the databases of Embase, Scopus, CENTRAL, Web of Science, and PubMed were searched from the inception of the databases to 30 January 2024. The primary outcome was overall survival (OS), and the secondary outcome was progression-free survival (PFS).

**Results:**

A total of 23 studies were available for review. A meta-analysis of 22 studies showed that high CONUT was significantly associated with poor OS in patients with hematological malignancies (HR: 1.95 95% CI: 1.62, 2.35 *I*^2^ = 89%). The results remained unchanged on sensitivity and subgroup analyses based on study location, sample size, diagnosis, CONUT cutoff, and the Newcastle–Ottawa Scale score. Only six studies reported data on PFS, and the pooled analysis found that high CONUT was a significant marker for poor PFS in patients with hematological malignancies [hazards ratio (HR): 1.64 95% CI: 1.21, 2.20 *I*^2^ = 70%]. These results, too, maintained significance in the sensitivity analysis.

**Conclusion:**

CONUT is an independent predictor of poor OS in patients with hematological malignancies. The results appear to be valid across different cancer types and with different CONUT cutoffs. Scarce data also suggest that CONUT could predict PFS.

## Introduction

Hematologic malignancies or blood cancers have become increasingly prevalent in the current century. These cancers are lymphatic and myeloid tumors caused by the disturbance of routine hematopoietic function and can be broadly classified into three types, namely, leukemias, myelomas, and lymphomas (1). Their incidence has peaked since the 1990s, with the global number of cases reaching 1,343,850 in 2019 (2). They account for approximately 10% of all cancer cases diagnosed in the USA, and their prevalence is projected to increase in the upcoming decade (1, 3). By 2030, there will be approximately 4,634,937 new cases of hematological malignancies, leading to significant mortality and disability in the affected individuals (3). Despite advances in chemotherapy and the discovery of newer drugs to manage such malignancies (4), survival remains poor and ranges from just 24 to 86% (5). Acute myeloid leukemia has the poorest 5-year survival rate of 24%, while Hodgkin lymphoma has the highest survival rate of 86% (5). Given the poor survival rate, it is necessary to recognize important prognostic indicators that can be suitably targeted to improve outcomes in such patients.

Malnutrition is highly prevalent among cancer patients across the world. Approximately 70% of all patients hospitalized for malignancies are under-nourished, and this accounts for approximately 20% of all cancer-related deaths (6). Among patients with hematological malignancies, approximately 17–43% are malnourished (7, 8). Indeed, malnutrition is frequently under-studied and under-investigated in clinical practice, despite being an important predictor of adverse outcomes. Malnutrition can reduce the response to anti-cancer therapy, worsen the probability of survival, increase recurrence, and prolong hospital stay (7, 9). One of the major limitations in the identification of malnutrition is the availability of a robust, easy-to-use, and reliable marker. The patient-generated Subjective Global Assessment and the Subjective Global Assessment (SGA) tools have been recommended for nutritional assessment in cancer patients owing to their high specificity and sensitivity compared to other tools. However, these assessments are time-consuming and difficult to complete even by well-trained professionals (10). Therefore, newer easy-to-use and rapid nutritional screening tools have been developed, such as the prognostic nutritional index, geriatric nutritional risk index, Mini-Nutrition Assessment (MNA), Malnutrition Universal Screening Tool (MUST), Nutrition Risk Screening 2002, and the controlling nutritional status score (CONUT) (11). Nevertheless, no single marker has been recognized as the gold standard, and these are dichotomies on the ability of these markers to accurately predict outcomes.

CONUT was first described by de Ulíbarri et al. (12) in 2005 as a rapid nutritional assessment tool for the routine screening of all inpatients. The score gives points for specific ranges of albumin, cholesterol, and total leukocyte counts, which are then combined to obtain the total CONUT score. The patients are then stratified into four levels based on the CONUT score: normal (0–1 points), mild (2–4 points), moderate (5–8 points), and severe (9–12 points), with each level indicating an increased degree of malnourishment. The original authors have noted that CONUT has a specificity and sensitivity of 85 and 92%, respectively, when compared with full nutritional assessment as the gold standard (12). It also has a high agreement with the SGA, which is the recommended tool for nutritional screening in cancer patients (13). Since CONUT is obtained from routinely available blood counts, it provides a rapid, simple, objective, efficient, and reliable assessment of the nutritional status of the patient in clinical practice. The simplicity of CONUT can be gaged when compared with other systematic nutritional indicators such as the SGA, MNA, and MUST, which require complex measurements such as anthropometry, dietary intake change, and weight loss history. The elaborate process in the latter tools is often time-consuming, difficult to complete, and not suitable for busy clinical practice (10).

In literature, CONUT has been used for predicting outcomes of colorectal (14), hepatic (15), gastric (16), bladder (17), and breast cancer (18). Furthermore, it has also been recognized as an independent risk factor for poor outcomes in patients with coronary artery disease (19), stroke (20), and hip fracture (21). Given its high validity, several studies have also examined the role of CONUT in predicting prognosis in hematological malignancies, but with variable results. In a previous study, Lu et al. (22) attempted to review the prognostic ability of CONUT for hematological malignancies but could include only six studies. We hereby present a comprehensive and updated review examining the prognostic ability of CONUT in predicting survival after hematological malignancies.

## Materials and methods

The reviewers abided by the Preferred Reporting Items for Systematic Reviews and Meta-Analyses (PRISMA) statement reporting guidelines while performing and presenting this review (23). Pre-registration was performed on the International Register of Systematic Reviews, PROSPERO (CRD42024506152).

### Literature retrieving

Articles were searched in electronic format in the databases of Embase, Scopus, CENTRAL, Web of Science, and PubMed by two reviewers independently. The search included all publications from the inception of the databases to 30 January 2024 and included all articles irrespective of the language. However, the search was restricted to human studies published in either full-text or abstract form.

The search terms used were, “leukemia, lymphoma, myeloma, myelodysplastic, myeloproliferative, CONUT, and controlling nutritional status. We generated search strings by combining these keywords with Boolean operators and used them across all databases (Supplementary Table S1). To supplement the search, we examined Google Scholar as a source for gray literature and also hand-searched references, including original articles and pertinent reviews.

### Eligibility criteria and selection of studies

The searched articles were examined against the following PECOS inclusion criteria:

Population (P): Patients with any type of hematological malignancy, regardless of the disease stage and treatment.

Exposure (E): Malnutrition was defined by a high CONUT score. The cutoff for high CONUT was not predefined and varied according to each included study.

Comparison (C): Normal nutrition was defined by a low CONUT score.

Outcomes (O): Overall survival (OS) was the primary outcome, while progression-free survival (PFS) was the secondary outcome. In general, OS is defined as the time from treatment to death due to any reason. PFS is defined as the time from the start of treatment to the first recurrence or disease progression. In the protocol, we had mentioned recurrence-free survival as the outcome. However, since the included studies reported PFS, recurrence-free survival was substituted by PFS.

Study type (S): Prospective or retrospective cohort studies.

The review excluded studies not on CONUT, studies that did not indicate relevant outcomes, editorials, review articles, and non-peer-reviewed articles. If there was an overlap of the sample between the two articles, we included the one with the largest sample size.

We initially combined the search results of all databases in a single reference manager software (EndNote). Articles were then deduplicated electronically. The two reviewers then carefully read the title and abstract of each study for initial screening. Relevant records were identified and their full-texts were retrieved. Selected studies underwent full-text analysis against the inclusion criteria. Differences between reviewers, if any, were solved by dialog.

### Risk of bias and data management

The quality of studies was tested by the Newcastle–Ottawa Scale (NOS) (24). Two reviewers were involved. Studies were judged for the criteria for participant selection, comparability of groups, and validity of results. The number of stars (ranging from 1 to 9) determined the study quality, with a higher number of stars indicating better quality.

Data on study information, patient demographic factors, CONUT cutoff, timing of measurement, percentage classified as malnourished, treatment, outcomes, and follow-up were recorded from the studies by two reviewers. Outcome data were also extracted separately and cross-checked for correctness. Multivariable adjusted ratios on OS and PFS were extracted. If unavailable, they were calculated from raw data. In cases where data were not provided, we attempted to contact the corresponding author once via email.

### Statistical analysis

All statistical analyses were performed using “Review Manager” (RevMan, version 5.3). The difference in OS and PFS between high and low CONUT groups was presented as hazard ratios (HRs) and 95% confidence intervals (CIs). Individual ratios from studies underwent logarithmical transformation using the generic inverse variance function of the Review Manager. Data were then combined in the inverse variance random-effect model. Publication bias was judged from funnel plots and the Egger’s test. Inter-study heterogeneity was checked by the chi-square-based Q statistics, and *I*^2^ statistics were used for inter-study heterogeneity. A *p*-value of < 0.10 for Q statistic, and *I*^2^ > 50% meant substantial heterogeneity.

Sensitivity and subgroup analyses were undertaken for the primary outcome. These analyses were not conducted for PFS due to the low number of studies. For the first analysis, individual studies were removed one at a time, and the HR was checked for significance. The subgroup analysis was performed based on the following variables: study location, sample size, diagnosis, CONUT cutoff, and NOS score.

## Results

**Figure 1:**
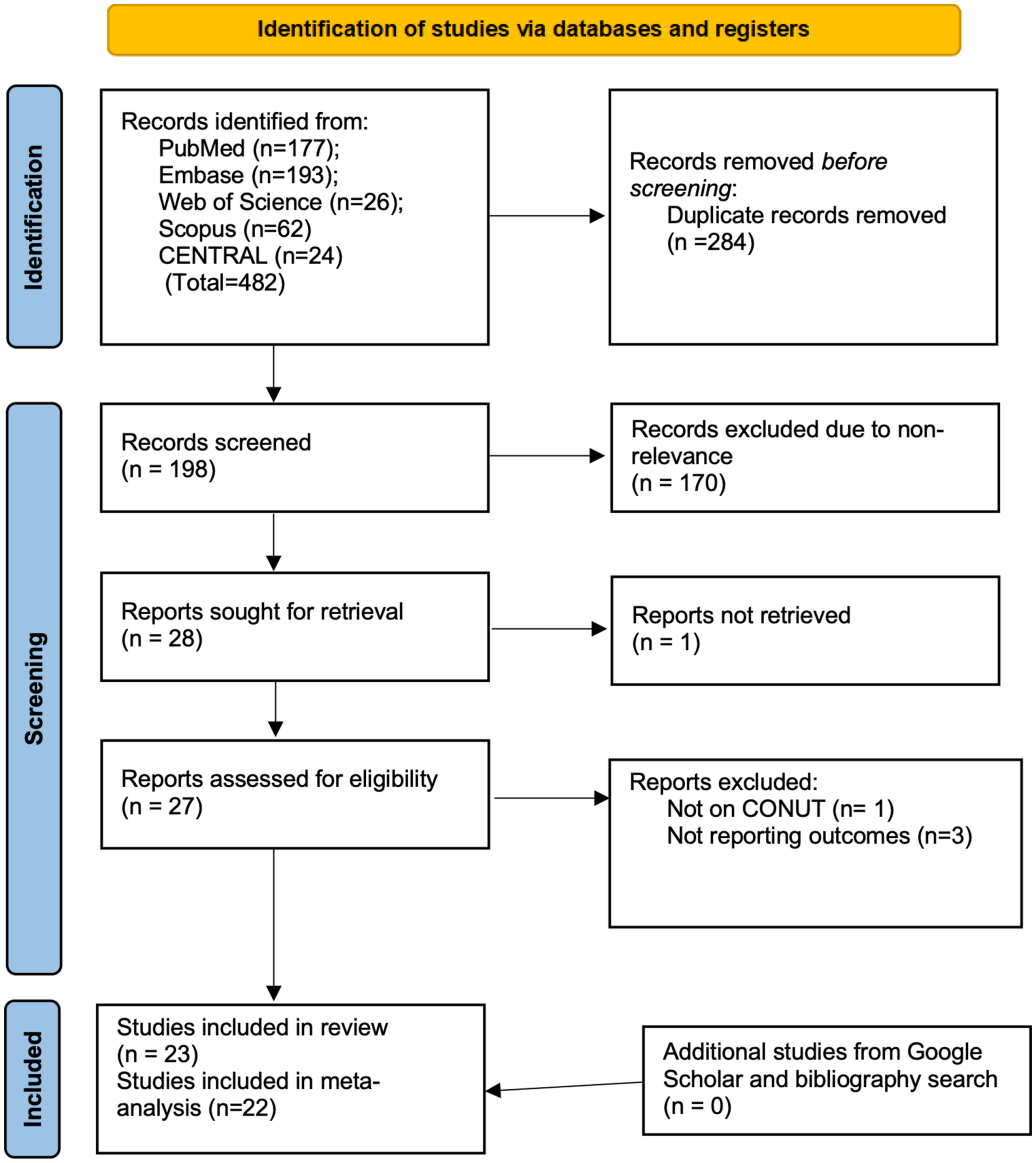
Search results of the review.

The PRISMA flowchart is presented in Figure 1. Of the 28 records that were selected for review, 1 could not be retrieved (25). Of the 27 remaining studies, 23 (26–48) were included in the review, and 4 were excluded for various reasons [1 was not on CONUT (49) and 3 did not report the required data (50–52)]. There were no disagreements among the reviewers regarding the inclusion or exclusion of any study. Furthermore, no additional study was identified from the gray literature and bibliography searches.

Data retrieved from studies are demonstrated in Table 1. All studies were from only three countries, namely, Japan (39.1%), China (43.5%), and Turkey (17.4%). All were retrospective cohort studies published between 2019 and 2023. Three studies did not report the mean/median age of patients. In the remaining studies, the age of the patients ranged from 41.6 to 75 years. The included patient populations were affected by myeloid malignancies and multiple myeloma (17.6%), adult T-cell leukemia and lymphoma, (0.9%), myelodysplastic syndromes and acute myeloid leukemia (5.7%), HIV-related lymphoma (2.7%), peripheral T-cell lymphoma (1.8%), Hodgkin lymphoma (5.6%), extranodal NK/T-cell lymphoma (28.2%), indolent non-Hodgkin lymphoma (1.9%), and diffuse large B-cell lymphoma (35.6%). The number of patients in the included studies ranged from 54 to 1,085. CONUT was measured either at diagnosis or at the start of treatment. The CONUT cutoffs used were 2, 3, 3.5, 4, 5, 5.5, and 6. Based on the respective cutoffs, the percentage of patients classified as malnourished ranged from 20.8 to 70.6. The included studies did not routinely report follow-up time. Where data were available, follow-up ranged from 10.5 months to 6.82 years. On quality assessment based on the NOS score, two studies received six stars while three got seven stars. The remaining studies received eight or nine stars, indicating good quality.

**Table 1:** Details extracted from included studies.

Author, year	Country	Study type	Population	*N*	Male (%)	Median age [IQR] or (range) (years)	CONUT cutoff	Timing of measurement	Mal-nourished (%)	Treatment	Outcomes	Follow-up (months)	NOS score
Araie et al. (26)	Japan	R	Myeloid malignancies	200	50.5	46 [18–67]	5	Before conditioning	28	Allo-HCT	NRM	44.6	8
Ureshino et al. (27)	Japan	R	Adult T-cell leukemia/lymphoma	54	51.9	NR	5	At diagnosis	35.2	Chemotherapy and Allo-HCT	OS	NR	6
Kamiya et al. (42)	Japan	R	Multiple myeloma	178	29.2	NR	5	NR	47.2	Protease inhibitors and immunomodulatory drugs	OS	NR	9
Matsukawa et al. (44)	Japan	R	DLBCL	615	54.8	69 [20–97]	4	NR	22.9	R-CHOP	OS	NR	8
Nagata et al. (43)	Japan	R	DLBCL	476	54.8	68.5 (27–97)	4	At diagnosis	31.3	R-CHOP	OS, PFS	45	8
Okamoto et al. (38)	Japan	R	Multiple myeloma	64	51.6	66 [41–84]	5	NR	28.1	Protease inhibitors, immunomodulatory drugs, and auto-PBSCT	OS	NR	9
Sakurai and Nakazato (45)	Japan	R	Myelodysplastic syndrome, acute myeloid leukemia	90	66.7	75 (43–93)	5	NR	37.8	Azacitidine	OS	10.5	8
Baysal et al. (48)	Turkey	R	DLBCL	81	51.9	63.5 ± 16.3*	5	NR	37	R-CHOP	OS	NR	8
Cagliyan et al. (29)	Turkey	R	DLBCL	266	50.8	68 (23–91)	2	At diagnosis	45.1	R-CHOP	OS, PFS	51	8
Li et al. (28)	China	R	Multiple myeloma	119	NR	56 [NR]	3.5	At diagnosis	NR	NR	OS	NR	7
Liang et al. (46)	China	R	Multiple myeloma	157	56.1	64 [NR]	3.5	NR	45.2	Chemotherapy	OS	24	8
Zhou et al. (47)	China	R	Multiple myeloma	245	59.2	65 (33–83)	4	NR	NR	Chemotherapy	OS	38	8
Chen et al. (33)	China	R	Myelodysplastic syndrome	121	68.6	65 [59–72]	4	At diagnosis	57.9	NR	OS	NR	7
Liu et al.(31)	China	R	HIV-related lymphoma	149	84.6	53.1 ± 15.1*	6	NR	20.8	Chemotherapy	OS	44	8
Nakamura et al. (32)	Japan	R	Peripheral T-cell lymphoma	99	69.7	67 (16–87)	5	At diagnosis	38.4	Chemotherapy and auto-PBSCT	OS	81.8	8
Qian et al. (30)	China	R	Myelodysplastic syndrome	81	56.8	64 (18–82)	5.5	At diagnosis	39.5	Chemotherapy	OS	13.1	7
Go et al. (37)	China	R	DLBCL	305	57.4	NR	5	Start of treatment	28.2	R-CHOP	OS, PFS	106	8
Gursoy et al. (40)	Turkey	R	Hodgkin lymphoma	307	56.4	41.6 ± 16.3*	3	NR	27.7	Chemotherapy	OS	63.4	6
Kaneda et al. (41)	Japan	R	DLBCL	203	59.1	74 (65–93)	3	At diagnosis	NR	R-CHOP	OS	48	8
Lu et al. (36)	China	R	Extranodal NK/T-cell lymphoma	80	72.5	51.5 [41.5, 60]	5	NR	NR	Chemotherapy and radiotherapy	OS, PFS	NR	8
Tiglioglu et al. (35)	Turkey	R	Indolent non-Hodgkin lymphoma	109	47.7	61.6 ± 12.8*	2	At diagnosis	52.3	Chemotherapy	OS, PFS	NR	8
Wu et al. (34)	China	R	Extranodal NK/T-cell lymphoma	374	65.0	44 (18–79)	2	Seven days prior to treatment	70.6	Chemotherapy and radiotherapy	OS, PFS	82	9
Zhang et al. (39)	China	R	Extranodal NK/T-cell lymphoma	1,085	47.3	47 [35–57]	NR	NR	NR	NR	OS, PFS	62.7	8

Allo-HCT, allogeneic hematopoietic stem cell transplantation; R, retrospective; P, prospective; NOS, Newcastle–Ottawa Scale; CONUT, controlling nutritional status; NRM, non-relapse mortality; DLBCL, diffuse large B-cell lymphoma; R-CHOP, rituximab, cyclophosphamide, doxorubicin, vincristine, and prednisone; auto-PBSCT, autologous peripheral blood stem cell transplantation; HIV, human immunodeficiency virus; N, number of participants; IQR, interquartile range.

*Mean ± standard deviation values.

**Figure 2:**
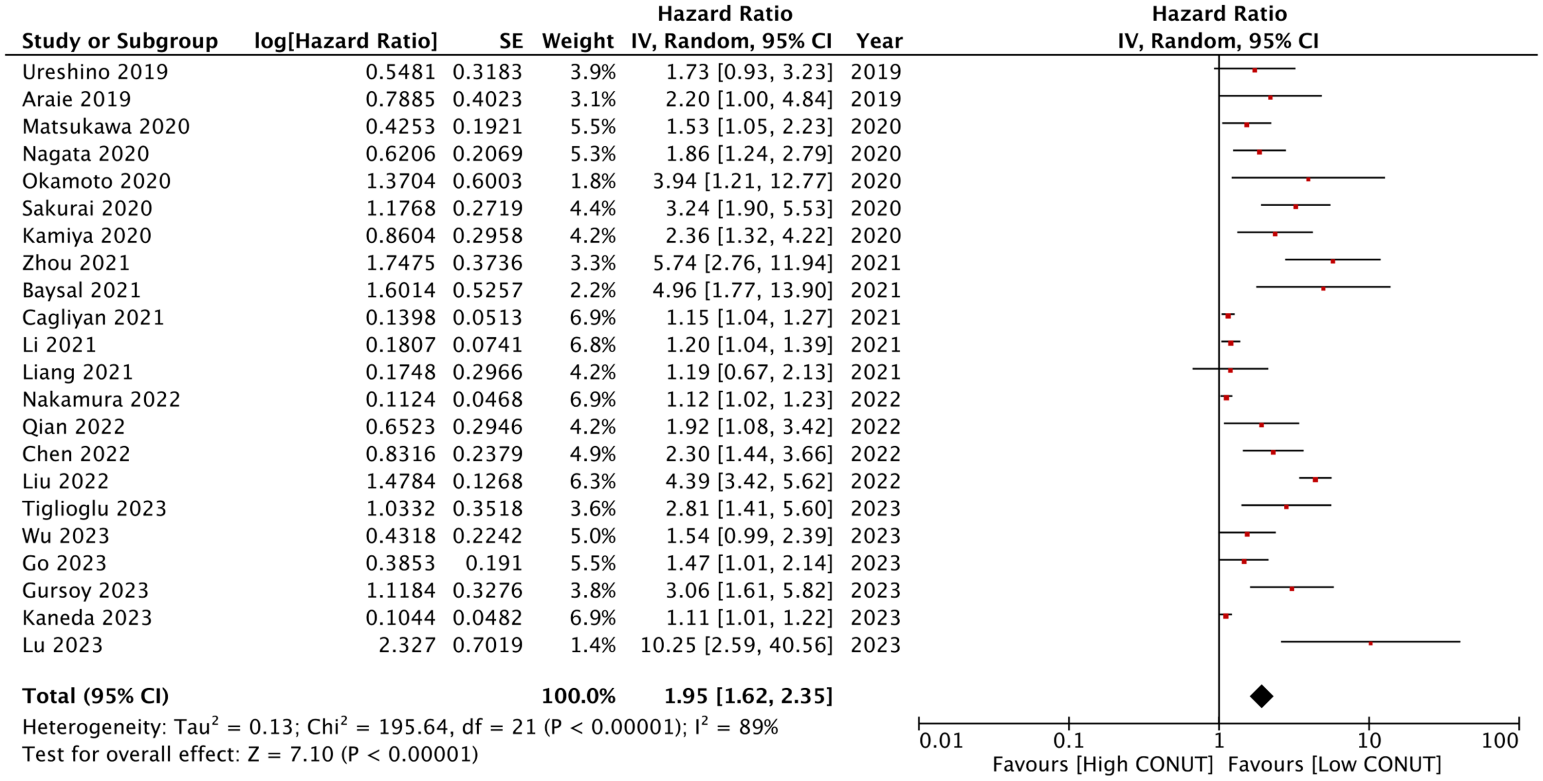
The meta-analysis of overall survival between high CONUT and low CONUT.

**Figure 3:**
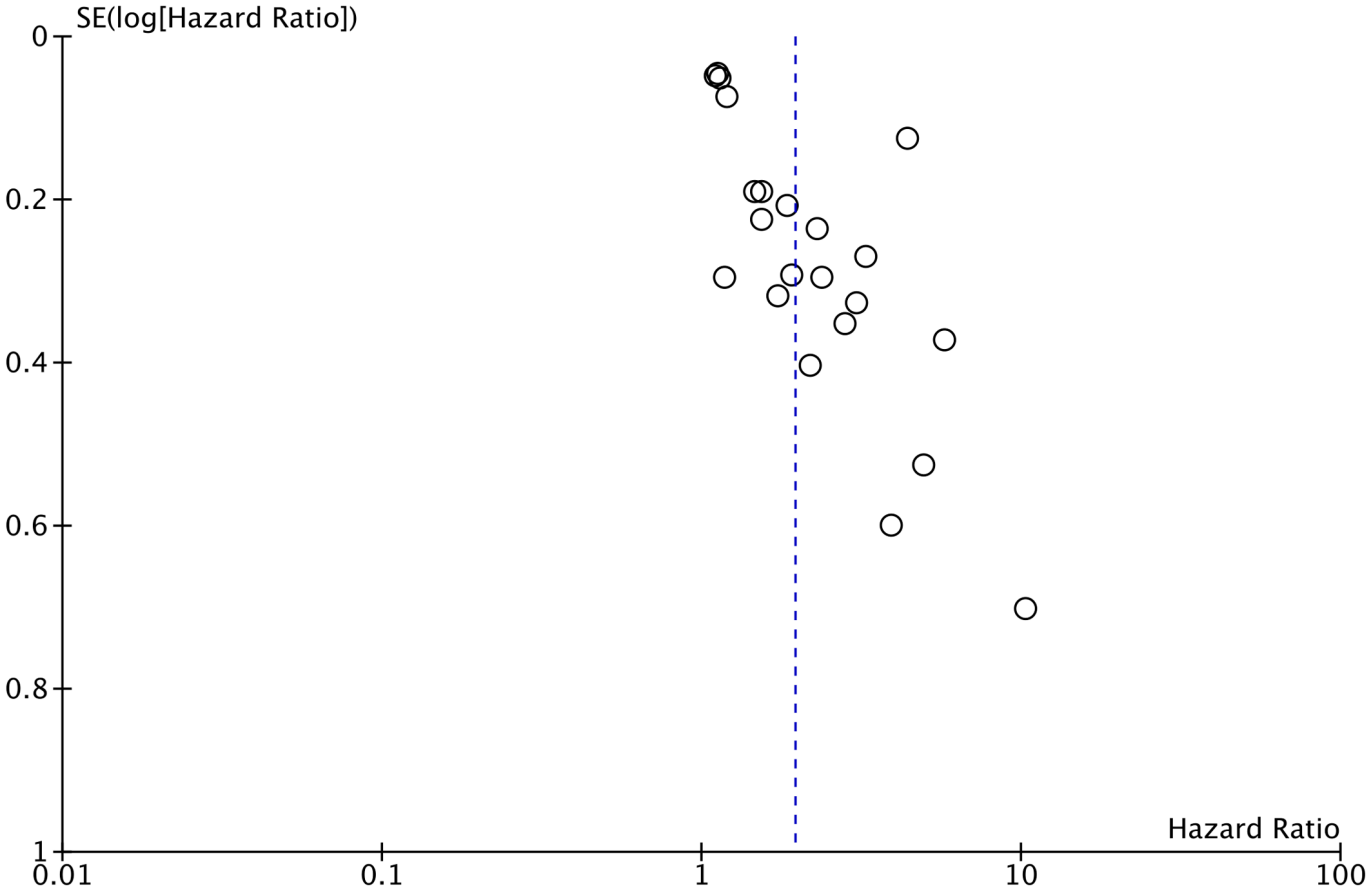
A funnel plot for judging publication bias.

Data for meta-analysis on OS were available from all studies. Zhang et al. (39) did not report HR based on a specific CONUT cutoff and used the score as a continuous variable. Hence, their study was not included in the meta-analysis. Using data from 22 studies, we found that high CONUT was significantly associated with poor OS in patients with hematological malignancies (HR: 1.95 95% CI: 1.62, 2.35 *I*^2^ = 89%) (Figure 2). The funnel plot is presented in Figure 3. There was no gross asymmetry noted on the funnel plot, and Egger’s test found no publication bias (*p* = 0.08). During the sequential exclusion of studies for the sensitivity analysis, we noted no change in the significance of the results. Multiple subgroup analyses were conducted for OS. Detailed results can be found in Table 2. There was no change in the significance of the results on subgroup analyses based on study location, sample size, diagnosis, CONUT cutoff, and NOS score.

**Table 2:** Subgroup analysis for overall survival.

Variable	Groups	Studies	Hazard ratio [95% confidence intervals]	*I* ^2^
Location	Turkey	4	2.44 [1.15, 5.13]	86
	China	9	2.26 [1.44, 3.56]	92
	Japan	9	1.60 [1.30, 1.97]	78
Diagnosis	Myeloid malignancies	6	2.15 [1.28, 3.62]	81
	Myelodysplastic syndrome and AML	3	2.44 [1.81, 3.30]	0
	DLBCL	6	1.34 [1.13, 1.59]	71
	Other lymphomas	6	2.69 [1.34, 5.41]	96
Sample size	>100	15	1.88 [1.49, 2.36]	91
	<100	7	2.60 [1.49, 4.56]	85
CONUT cutoff	2–3.5	7	1.27 [1.10, 1.47]	66
	4	4	2.27 [1.47, 3.52]	71
	5–6	11	2.56 [1.61, 4.08]	93
NOS score	8–9	17	2.01 [1.61, 2.51]	91
	6–7	5	1.85 [1.25, 2.76]	5

AML, Acute myeloid leukemia; DLBCL, diffuse large B-cell lymphoma.

**Figure 4:**
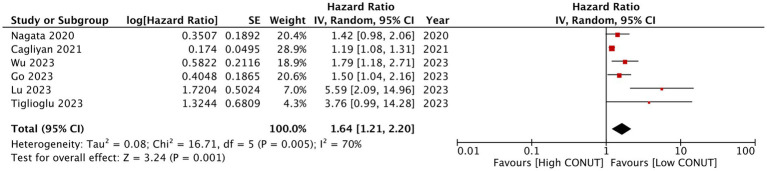
The meta-analysis of progression-free survival between high CONUT and low CONUT.

A meta-analysis of PFS is presented in Figure 4. Only six studies reported data on PFS, and the pooled analysis found that high CONUT was a significant marker for poor PFS in patients with hematological malignancies (HR: 1.64 95% CI: 1.21, 2.20 *I*^2^ = 70%). The pooled effect size of PFS remained significant in the sensitivity analysis.

## Discussion

The present review accounts for the most updated evidence on the prognostic role of CONUT in patients with hematological malignancies. We combined data from a large number of studies to show that high CONUT was a significant predictor of poor OS. The negative effect of CONUT on OS was persistent, even with various subgroups and sensitivity analyses. Similarly, the quantitative analysis from a much smaller number of studies also demonstrated that patients with high CONUT have poor PFS.

The validity of CONUT in predicting survival in cancer patients is well-established. Takagi et al. (14) in a meta-analysis of nine studies have found CONUT to be a predictor of OS (HR: 1.97), PFS (HR: 1.68), and cancer-specific survival (HR: 3.64) in colorectal cancer. CONUT has been found to independently predict OS (HR: 1.78), PFS (HR: 1.34), and postoperative complications (HR: 1.85) in hepatocellular cancer patients undergoing surgical intervention (15). Another meta-analysis of five studies has noted that high CONUT is associated with poor OS (HR: 1.78), PFS (1.34), and postoperative complications (HR: 1.85) in gastric cancer patients undergoing gastrectomy (16). The results of our review further expand the validity of CONUT in patients with hematological malignancies. Using a broad search strategy without any limitations of language or publication dates, we were able to retrieve and analyze 23 studies examining the association between CONUT and survival after hematological malignancies. A combined analysis of 22 studies showed that high CONUT led to worse OS, increasing the risk of mortality by 92%. The consistent positive association between CONUT and poor OS among the included studies and the stability of the results on sensitivity analysis lend credibility to CONUT as a rapid prognostic indicator for clinical practice. Nevertheless, the high inter-study heterogeneity is a cause for concern. This can be partially attributed to the variations in the included populations, the variable CONUT cutoffs, and the treatment offered to the included patients. Hematological malignancies include a large group of disorders such as multiple myeloma, lymphomas, leukemias, and myelodysplastic syndromes, and CONUT might have different predictability in different malignancies. However, in the multiple subgroup analyses conducted, CONUT was consistently associated with poor OS in patients with myeloid malignancies, myelodysplastic syndromes, diffuse large B-cell lymphoma, and other lymphomas. Similarly, the location of studies, sample size, and quality of studies also did not affect the significance of the association. While the majority of studies reported data on OS, only six reported on PFS. The meta-analysis found that high CONUT was associated with worse PFS in hematological malignancies. Similar to OS, the results were more or less consistent across studies and stable on the sensitivity analysis.

One major inconsistency noted among the included studies was the cutoff of CONUT, which defined malnutrition. As mentioned earlier, CONUT has different levels of malnutrition ranging from mild to severe, but these may not be consistently replicable across all populations. Some studies use the standard cutoffs of CONUT and define malnutrition as those with scores of >5 (moderate–severe CONUT scores), while others use the receiver operator characteristic curve to establish the best cutoff in their population. Such variability has been noted in the earlier meta-analyses (14–16) as well and has been a major hindrance to the applicability of CONUT in clinical practice. In our subgroup analysis based on different CONUT cutoffs, we noted that all cutoffs of CONUT were associated with poor OS. However, the effect size increased with higher cutoffs. The HRs for cutoffs of 2–3.5, 4, and 5–6 were 1.27, 2.27, and 2.56, respectively, indicating that an incremental increase in malnutrition is associated with worse OS.

The predictive value of CONUT for survival outcomes can be attributed to its three components. Albumin is an essential marker of the nutritional and immune status of the patient. Malnourished patients also have poor responses to chemotherapy and increased drug-related toxicity, which is an important factor in treating hematological malignancies where chemotherapy is the primary therapeutic modality (53). Furthermore, poor nutrition has been associated with depression, which can be a major hindrance to treatment and therefore worsens survival (54). Albumin is also a marker for the systemic inflammatory response in cancer patients (53). Individually, serum albumin has been shown to be an independent marker of mortality in lymphoma and leukemia patients (55, 56). Second, lymphocytes are the primary component of cell-mediated immunity, as they can control cancer proliferation and metastasis, promote apoptosis, and play an important role in immune surveillance (34). Reduced lymphocyte counts have also been associated with worse clinical features in hematological malignancies (57). Finally, cholesterol plays a major role in maintaining the integrity of cell membranes and immunity, which ensures that immunocompetent cells can attack cancer cells (58). Gao et al. (59) have shown that diffuse large B-cell lymphoma patients with hypocholesterolemia have a worse clinical presentation, such as a high International Prognostic Index score, B symptoms, and advanced stage, as compared to those with normal cholesterol levels. Thus, it can be summarized that all three components of CONUT are associated with cancer survival, and, when combined, they produce a robust easy-to-use marker.

The important strength of the review includes a detailed literature search without any barriers to language or sample size. Our study provides the most comprehensive evidence on the ability of CONUT to predict survival in hematological malignancies. In comparison with the previous review by Lu et al. (22), we added 17 more studies, thereby increasing the statistical power of the meta-analysis. We also undertook sensitivity analyses and detailed subgroup analyses to provide clarity on the topic.

However, several limitations still exist. None of the included studies were prospective, and therefore, they were prone to bias. Another aspect to consider is the high inter-study heterogeneity. Despite detailed subgroup analyses, much heterogeneity persisted in the meta-analysis and could be due to the differences in patient populations, cancer stage, and treatment provided. We were unable to examine the influence of important moderators such as body mass index, cancer stage, CNS/liver/bone marrow invasion, and bone marrow transplantation on survival outcomes due to a lack of data. Second, there were limited data on PFS and other outcomes, such as treatment response and treatment complications. The latter could not be included in the meta-analysis. Third, all of the data were from only three countries. There was a lack of data in the literature from Western populations, and hence, the results cannot be generalized. At this point, we could not find any concrete reasons for the non-reporting of CONUT from Western countries. Given the ease of use of CONUT, there should not be any reason for not applying CONUT in clinical practice, even in Western populations. We believe that the results of this study may prompt further reporting of data from Western countries and, hence, improve evidence. Fourth, we included myelodysplastic syndromes in the meta-analysis to ensure completeness. Myelodysplastic syndromes frequently lead to acute myeloid leukemia, and they have been considered a type of malignancy by the American Cancer Society. The inclusion of these populations may also have led to heterogeneity in the meta-analysis. Finally, we examined the impact of only pretreatment CONUT scores on OS/PFS. There are no data in the literature on how changes in CONUT scores affect outcomes or how nutritional interventions affect CONUT scores. This could be a topic of research in future studies, and such evidence could help personalize treatment plans, which in turn could improve outcomes in hematological malignancies.

## Conclusion

CONUT is an independent predictor of poor OS in patients with hematological malignancies. The results appear to be valid across different cancer types and with different CONUT cutoffs. Scarce data also suggest that CONUT could predict PFS. There is a need for more data from Western populations on the impact of CONUT on other outcomes, such as PFS and treatment response.

## Author contributions

GL: Conceptualization, Data curation, Formal analysis, Investigation, Methodology, Software, Writing – original draft. QL: Data curation, Formal analysis, Investigation, Methodology, Project administration, Software, Supervision, Validation, Visualization, Writing – review & editing.
