# Supplementary material for: The controlling nutritional status score as a predictor of survival in hematological malignancies: a systematic review and meta-analysis
Source: Front Nutr. 2024 Jun 13;11:1402328. doi: 10.3389/fnut.2024.1402328 (PMC11208478; doi:10.3389/fnut.2024.1402328)
Supplement: Supplementary file 1 [file Table_1.DOCX]

**Supplementary Table-1**. Methodology of search

| **Key words** | **Search Details** |
| --- | --- |
| (((((Leukemia) OR (lymphoma)) OR (myeloma)) OR (myelodysplastic)) OR (myeloproliferative)) AND (CONUT) | ("leukaemia"[All Fields] OR "leukemia"[MeSH Terms] OR "leukemia"[All Fields] OR "leukaemias"[All Fields] OR "leukemias"[All Fields] OR "leukemia s"[All Fields] OR ("lymphoma"[MeSH Terms] OR "lymphoma"[All Fields] OR "lymphomas"[All Fields] OR "lymphoma s"[All Fields]) OR ("multiple myeloma"[MeSH Terms] OR ("multiple"[All Fields] AND "myeloma"[All Fields]) OR "multiple myeloma"[All Fields] OR "myeloma"[All Fields] OR "myelomas"[All Fields] OR "myeloma s"[All Fields]) OR ("myelodysplastic"[All Fields] OR "myelodysplastics"[All Fields]) OR "myeloproliferative"[All Fields]) AND "CONUT"[All Fields] |
| (((((Leukemia) OR (lymphoma)) OR (myeloma)) OR (myelodysplastic)) OR (myeloproliferative)) AND (controlling nutritional status) | ("leukaemia"[All Fields] OR "leukemia"[MeSH Terms] OR "leukemia"[All Fields] OR "leukaemias"[All Fields] OR "leukemias"[All Fields] OR "leukemia s"[All Fields] OR ("lymphoma"[MeSH Terms] OR "lymphoma"[All Fields] OR "lymphomas"[All Fields] OR "lymphoma s"[All Fields]) OR ("multiple myeloma"[MeSH Terms] OR ("multiple"[All Fields] AND "myeloma"[All Fields]) OR "multiple myeloma"[All Fields] OR "myeloma"[All Fields] OR "myelomas"[All Fields] OR "myeloma s"[All Fields]) OR ("myelodysplastic"[All Fields] OR "myelodysplastics"[All Fields]) OR "myeloproliferative"[All Fields]) AND (("controling"[All Fields] OR "controllability"[All Fields] OR "controllable"[All Fields] OR "controllably"[All Fields] OR "controller"[All Fields] OR "controller s"[All Fields] OR "controllers"[All Fields] OR "controlling"[All Fields] OR "controls"[All Fields] OR "prevention and control"[MeSH Subheading] OR ("prevention"[All Fields] AND "control"[All Fields]) OR "prevention and control"[All Fields] OR "control"[All Fields] OR "control groups"[MeSH Terms] OR ("control"[All Fields] AND "groups"[All Fields]) OR "control groups"[All Fields]) AND ("nutritional status"[MeSH Terms] OR ("nutritional"[All Fields] AND "status"[All Fields]) OR "nutritional status"[All Fields])) |
